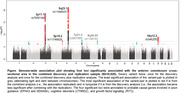# The genetic architecture of the human anterior commissure and its potential role as biomarker of neurodegeneration

**DOI:** 10.1002/alz.085587

**Published:** 2025-01-09

**Authors:** Carolina Ochoa‐Rosales, Hieab H.H. Adams

**Affiliations:** ^1^ BrainLat, Universidad Adolfo Ibañez, Santiago, Region Metropolitana Chile; ^2^ Radboud University Medical Center, Nijmegen, Gelderland Netherlands; ^3^ Latin American Brain Health (BrainLat), Universidad Adolfo Ibáñez, Santiago, Santiago Chile

## Abstract

**Background:**

Commissural tracts are the white matter fibre bundles intercommunicating left and right brain hemispheres. They integrate many cognitive functions such as memory, verbal processing, motor and perceptual skills. Also, commissures connect specific layers of cortical neurons that are also lost in Alzheimer’s disease (AD) and other neurodegenerative disorders. Although highly heritable, commissures´ specific genetic determinants remain obscure. We aim to investigate the genetic determinants of the human anterior commissure. Given its presumed role in neurodegeneration, we aim to further provide mechanistic insights into neurological conditions that may result from its dysfunction.

**Method:**

Two‐stage genome‐wide association study, (GWAS) (N=18,828) of the size of the anterior commissure. The discovery sample included seven cohorts (N=7,935) and was meta‐analyzed with ten replication cohorts (N=10,893). The size of the anterior commissure was manually derived from magnetic resonance imaging (1.5T/3T) with at least T1/T2‐weighted sequences. The genetic data was assessed through genotyping using SNP microarrays. We used voxel‐based morphometry to determine which regions are connected by the anterior commissure. To prove a functional validation of the identified variants, we performed a series of *in silico* experiments, including the study of the spatial expression patterns in human brains, quantitative trait loci (QTL), enrichment analysis and pleiotropy with neurodegenerative diseases.

**Result:**

we identified six independent variants at four loci (p‐values from 4.1x10‐8 to 9.4x10‐22). We mapped the loci to probable causal genes involved in axon guidance (*EPHA3* and *SEMA6A*), cognitive disorders (*CTNND2*), and growth factor signaling (*RIT2*). Voxel‐based morphometry revealed distinct associations of the variants with connected grey matter regions in the brain. We found enrichment for H3K4me1 peaks (marking enhancer sites), introns, and conserved sequences, as well as cell‐type‐specific annotations from the central nervous system and cardiovascular system. Furthermore, we identified pleiotropy between genes known to increase risk of neurodegenerative conditions including frontotemporal lobar degeneration gene *TMEM106B*. Variants associated to this gene have been related to dementia development.

**Conclusion:**

these results shed light on the genetic architecture of commissural tracts and establish the size of the anterior commissure as a relevant biomarker of neurodegeneration.